# Stroke Lesion Segmentation in FLAIR MRI Datasets Using Customized Markov Random Fields

**DOI:** 10.3389/fneur.2019.00541

**Published:** 2019-05-24

**Authors:** Nagesh K. Subbanna, Deepthi Rajashekar, Bastian Cheng, Götz Thomalla, Jens Fiehler, Tal Arbel, Nils D. Forkert

**Affiliations:** ^1^Department of Radiology, University of Calgary, Calgary, AB, Canada; ^2^Department of Neurology, University Medical Center Hamburg-Eppendorf, Hamburg, Germany; ^3^Department of Neuroradiology, University Medical Center Hamburg-Eppendorf, Hamburg, Germany; ^4^Centre for Intelligent Machines, McGill University, Montreal, QC, Canada

**Keywords:** magnetic resonance imaging, ischemic stroke, image segmentation, classification, brain lesion segmentation

## Abstract

Robust and reliable stroke lesion segmentation is a crucial step toward employing lesion volume as an independent endpoint for randomized trials. The aim of this work was to develop and evaluate a novel method to segment sub-acute ischemic stroke lesions from fluid-attenuated inversion recovery (FLAIR) magnetic resonance imaging (MRI) datasets. After preprocessing of the datasets, a Bayesian technique based on Gabor textures extracted from the FLAIR signal intensities is utilized to generate a first estimate of the lesion segmentation. Using this initial segmentation, a customized voxel-level Markov random field model based on intensity as well as Gabor texture features is employed to refine the stroke lesion segmentation. The proposed method was developed and evaluated based on 151 multi-center datasets from three different databases using a leave-one-patient-out validation approach. The comparison of the automatically segmented stroke lesions with manual ground truth segmentation revealed an average Dice coefficient of 0.582, which is in the upper range of previously presented lesion segmentation methods using multi-modal MRI datasets. Furthermore, the results obtained by the proposed technique are superior compared to the results obtained by two methods based on convolutional neural networks and three phase level-sets, respectively, which performed best in the ISLES 2015 challenge using multi-modal imaging datasets. The results of the quantitative evaluation suggest that the proposed method leads to robust lesion segmentation results using FLAIR MRI datasets only as a follow-up sequence.

## 1. Introduction

Strokes are a leading cause of death and disability worldwide (1). Acute ischemic strokes, which are caused by a blockade of an artery, account for 80% of all strokes. However, a blockade of an artery by a blood clot does not lead to an immediate necrosis of the brain region supplied by the blocked artery since collateral blood vessel connections can partly compensate the blocked blood flow from the main artery (2). It is typically assumed that the infarct core will gradually expand into this hypoperfused brain region over time if the blood clot is not dissolved (3). This region is typically referred to as the penumbra or tissue-at-risk (4–6) and represents the target for any ischemic stroke treatment.

For almost 25 years, the clot dissolving tissue plasminogen activator (tPA) was the only available treatment option with varying success rates depending on the clot location and morphology (7). Recent prospective randomized trials have shown overwhelming success of mechanical thrombectomy (8). Currently, more and more devices for mechanical thrombectomy are developed while at the same time more research is focusing on the development and evaluation of novel treatment approaches such as the use of neuroprotective drugs. In any case, clinical studies are required to show the efficacy of these new devices or treatment options. Although the clinical outcome (e.g., modified Rankin scale at 90 days post stroke) is typically used as the primary endpoint for such studies, the follow-up stroke lesion volume is becoming more important as an alternative primary or secondary study endpoint nowadays. This is not only because the continuous lesion volume has a higher statistical power compared to categorical outcome measures but also because it can be measured at an earlier time point.

**Figure 1:**
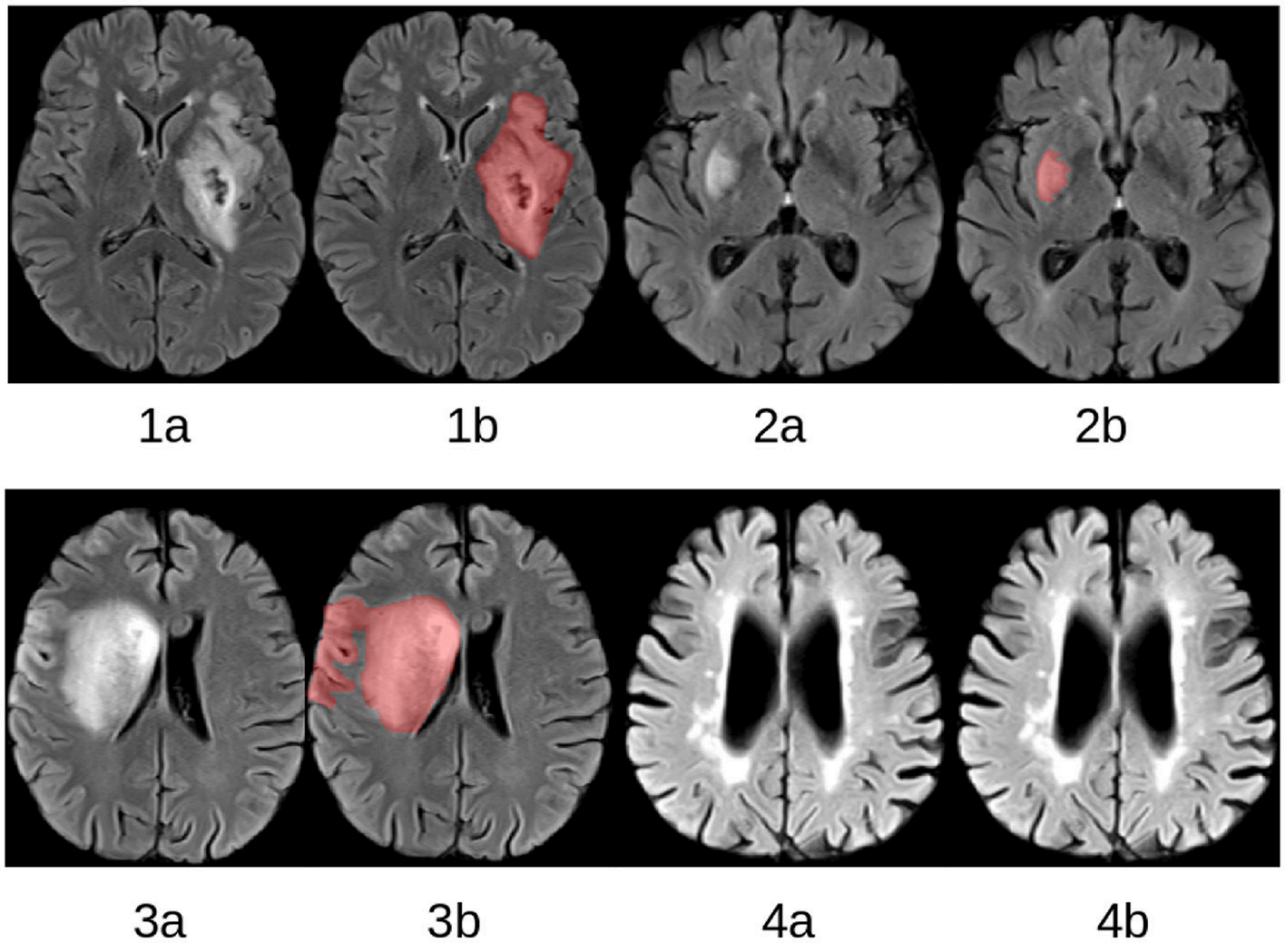
Selected slices from four FLAIR MRI datasets **(1a–4a)** with corresponding expert lesion segmentations **(1b–4b)**.The lesions vary considerably with respect to shape, position, and size. Patient 4 does not display a lesion resulting from an acute ischemic stroke but considerable white matter hyperintensities, which are often falsely segmented by automatic lesion segmentation methods.

Magnetic resonance imaging is one of the most used techniques for follow-up brain lesion assessment. However, the quantitative measurement of the lesion volume in follow-up imaging data requires a precise segmentation, which is a tedious and complex task, if performed manually, since stroke lesions vary considerably in shape, size, and location in the brain (see Figure 1).

Due to the importance of follow-up lesion segmentation, multiple methods for semi-automatic or automatic stroke lesion segmentation have been presented in the past, for example using unsupervised k-means clustering (9), active learning approaches (10), or more advanced extra tree forests (11), Markov random field (MRF) models (12), and convolutional neural networks (13). Almost all recently proposed lesion segmentation techniques utilize local features and contexts that can be affected by signal noise, geometric distortions, magnetization inhomogeneities, and anatomical variations. Furthermore, previously described methods often suffer from normalization problems inherent to non-quantitative imaging methods, which is especially important in case of multi-center datasets that are typically acquired using varying imaging parameters. Finally, if multiple imaging modalities with complementary information are used for lesion segmentation, a registration of all multi-modal datasets into a common reference space is required, which leads to interpolation artifacts but might also require extensive tuning of registration methods to account for all possible problems such as local imaging distortions, for example, often seen in case of diffusion-weighted MRI datasets acquired using an echo-planar imaging technique.

Fluid-attenuated inversion recovery (FLAIR) MRI (see Figure 1) is an image sequence that is typically used as a stroke follow-up sequence. Although using only FLAIR datasets for lesion segmentation does not leverage the multi-modal information, should it be available, it represents the lowest common denominator of image sequences typically available in follow-up stroke MRI protocols. Thus, it is useful to develop and evaluate lesion segmentation methods specifically and only for this image sequence, especially if historical datasets are used for the control group in a clinical trial.

A major issue for segmenting stroke lesions using FLAIR datasets only is that small white matter lesions, which are not part of the ischemic stroke lesion (see Figure 1), are often falsely segmented. In order to overcome this problem, we propose a technique based on MRFs that utilize multiple local and regional observations to improve the automatic stroke lesion segmentation using only FLAIR MRI datasets. Utilizing multiple observations, especially including texture features, often leads to more robust segmentation results. Similar techniques have been successfully developed and evaluated for other segmentation tasks such as the delineation of brain tumors (14) and multiple sclerosis lesions (15). In this paper, the generic graphical model framework described in (16) is adapted to segment stroke lesions by merging the two hierarchical levels into a single MRF model that includes local observations such as signal intensities and intensity variations, and regional observations such as texture features.

## 2. Materials and Methods

### 2.1. Material

Three different multi-center databases were used in this work for the development and evaluation of the proposed lesion segmentation method. All datasets were made available fully anonymized for this retrospective, secondary study so that ethics approval was not required.

The first database consists of 23 FLAIR MRI datasets acquired at the University Medical Center Hamburg-Eppendorf, Germany, for a clinical study focusing on the analysis, modeling, and modulation of the human motor network during recovery from motor stroke (17), which is referred to as the SFB database in the following. All of these datasets were acquired on a 3T Siemens Skyra MRI scanner (Siemens, Erlangen, Germany) using a 32-channel head coil (repetition time (TR) = 9,000 ms, echo time (TE) = 90 ms, inversion time (TI) = 2,500 ms, field of view = 230 × 230 mm^2^, slice thickness = 5 mm, and in-plane resolution = 0.7 × 0.7 mm^2^).

The second database consists of 108 FLAIR datasets acquired within a prospective European multi-center stroke imaging study (I-KNOW) from a total of five contributing centers (18). The aim of I-KNOW was to collect a large sample of patients with anterior circulation stroke, with admission and follow-up MRI to develop infarct prediction models based on clinical and imaging variables. The FLAIR imaging parameters and scanners (all 1.5T) used for image acquisition were different for all contributing centers. The slice thickness of these datasets ranges from 5.5 to 7.2 mm, while the in-plane resolution ranges from 0.45 × 0.45 mm^2^ to 0.94 × 0.94 mm^2^.

The third database consists of 28 FLAIR MRI datasets that are publicly available for classifier training as part of the Ischemic Stroke Lesion Segmentation (ISLES) challenge organized in conjunction with the Medical Image Computing and Computer Assisted Intervention (MICCAI) conference 2015 (19). These FLAIR datasets were acquired in two centers, which were both equipped with a 3T MRI scanner. Datasets of patients with isolated brainstem lesions were excluded from the original ISLES training database to ensure consistency with the other two databases. It also has to be highlighted that the FLAIR MRI datasets from this database were only available registered and resampled to the corresponding high-resolution T1-weighted MRI dataset and not as the original images. The in-slice spatial resolution of these registered images is 1.0 × 1.0 mm^2^ while the slice thickness is 1.0 mm in all cases.

Thus, a total of 159 FLAIR datasets of patients with an ischemic stroke acquired at the sub-acute phase (2–7 days post stroke onset) were available for this work. An experienced observer segmented all lesions in the first two databases using the in-house developed software tool AnToNIa (20). The procedure used for the manual lesion segmentation is described in detail in Cheng et al. (18). For the ISLES database, the ground truth segmentations available for the training datasets were used directly for the development and evaluation of the method described in this work.

### 2.2. Preprocessing

The datasets from the ISLES database are only available already skull stripped, which means that only brain tissue is visible in these datasets. As this was not the case for the other two databases available for this work, skull stripping was performed to ensure consistency between the databases. Therefore, the GIN-IMN elderly brain atlas (21) was skull stripped using the Brain Extraction Tool (BET) (22). After this, the GIN-IMN elderly brain atlas (21) was registered non-linearly to each FLAIR MRI dataset using ANTs (23) and the corresponding brain atlas segmentation was transformed accordingly to the each FLAIR dataset using Lanczos windowed sinc interpolation, which was then used for skull stripping.

As described above, the datasets used in this study were acquired using a variety of MRI scanners with different field strengths and imaging parameters. Thus, the FLAIR image intensities are not directly comparable between the different centers. To overcome this problem, an inter-patient intensity normalization was performed using image intensity histogram quartiles as control points as described in Nyl et al. (24).

After skull stripping, a non-uniformity intensity correction was performed using the N3 algorithm (25) to account for local bias field inhomogeneities. As high-intensity lesions in the FLAIR MRI datasets can negatively influence the calculation of the bias field, this computation was restricted to a brain mask that excluded high-intensity regions as well as the background. Practically, the mask was generated using a lower threshold of 0.12 and an upper threshold of 0.65 of the normalized FLAIR MRI datasets. These threshold values were manually selected because they led to suitable results across multiple different datasets. The bias field corrected FLAIR datasets were manually examined and datasets that showed artifacts due to the bias field correction were excluded from this study.

In the last step, the non-linear GIN-IMN elderly brain atlas to FLAIR MRI transformation as described above was also used to transform tissue prior maps of gray matter, white matter, and cerebrospinal fluid to each FLAIR MRI dataset.

### 2.3. Lesion Segmentation

**Figure 2:**
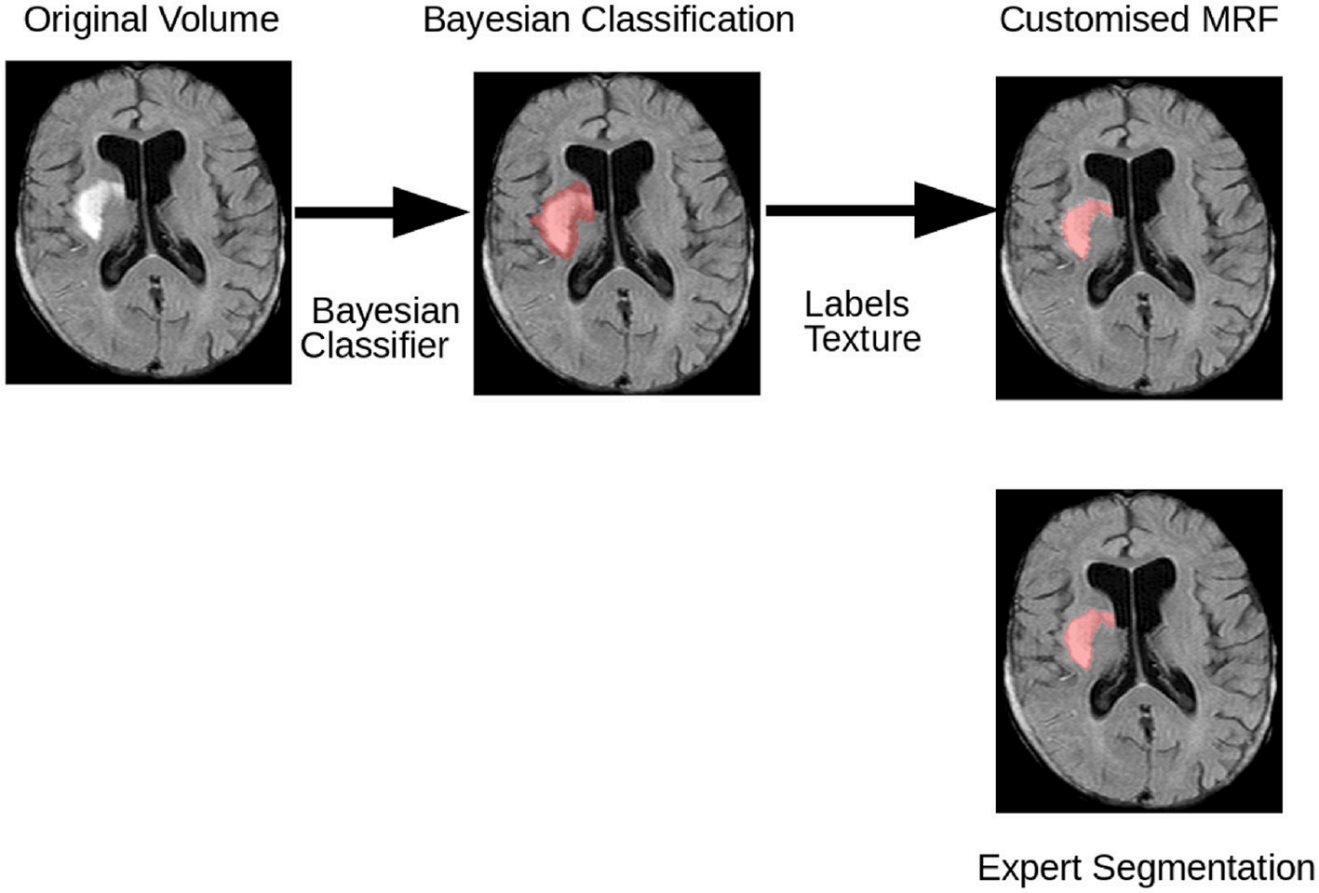
Flowchart illustrating the various stages of the method employed to segment stroke lesions. Initially, a Bayesian classifier is employed to classify each voxel of the preprocessed FLAIR MRI dataset into lesion and non-lesion voxels, based on the maximum a posteriori probability of the Gabor textures. Then, the customized voxel-based MRF utilizing the intensity of each voxel, intensities of the neighboring voxels, and the texture is used to compute the final classification.

The proposed method for lesion segmentation in FLAIR MRI datasets uses a two-step technique. In the first step, a Bayesian classification based on Gabor texture information is used to evaluate the probability of each voxel belonging to one of two groups, lesion or non-lesion voxels. The output of the Bayesian segmentation is then used as the input to a customized MRF, where each brain voxel is assigned one of *M* classes. In this work *M* = 4 classes are used, which include cerebrospinal fluid (CSF), gray matter (GM), white matter (WM), and the stroke lesion (Les). Figure 2 illustrates the processing steps of this two-step lesion segmentation method for automatic lesion delineation in FLAIR MRI datasets (only the lesion segmentation class is shown for the MRF output), described in more detail in the following.

#### 2.3.1. Initial Classification

For the initial Bayesian classification, each FLAIR dataset is decomposed into its multi-window Gabor filter bank outputs, **I**^*G*^, using the convolution at each voxel as described below. The class of each voxel, *C*_*i*_, is then estimated using Bayesian classification:

(1) $$P(C_{i}|I_{i}^{G})\propto P(I_{i}^{G}|C_{i})P(C_{i}),$$

where ${\text{I}}_{i}^{G}$ is the set of Gabor coefficients of the i*th* voxel. The likelihood term encapsulates the probability of the class, given the texture features in Gabor space. The technique to compute the Gabor features is described in the following.

#### 2.3.2. Gabor Window Computation

**Figure 3:**
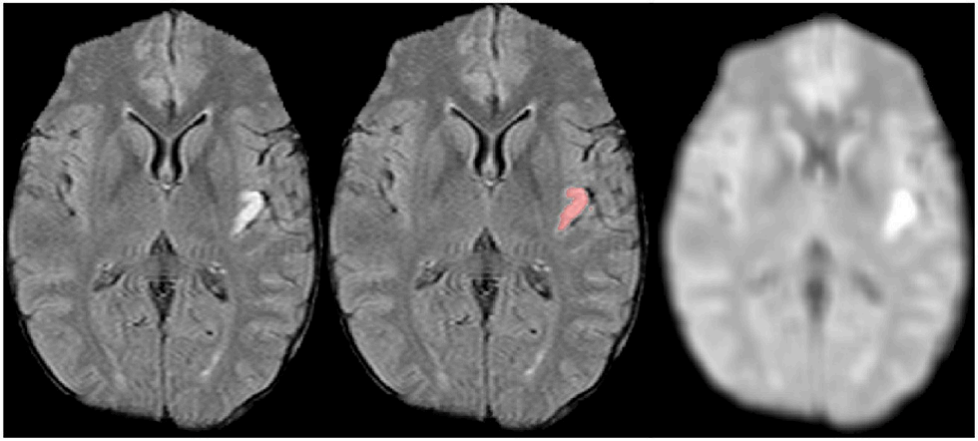
FLAIR MRI dataset **(Left)** with corresponding lesion segmentation **(Center)** and dominant Gabor texture coefficient image **(Right)**. Since the lesion is rather small, the middle (second octave) component Gabor function at 45° orientation to the x-axis dominates.

In a first step, each axial slice of a given FLAIR image is processed using multi-window, 2D discrete Gabor transforms as suggested in Zibulski and Zeevi (26). 2D instead of 3D Gabor transforms are used in this work since FLAIR MRI datasets usually exhibit a rather coarse slice thickness that is considerably worse compared to the in-slice spatial resolution. The set of R window functions is computed as suggested in Subbanna et al. (16):

(2) $$\begin{array}{l} g_{r}[x,y;a,b,n_{1},n_{2},m_{1},m_{2},\sigma_{x_{r}},\sigma_{y_{r}}]\\ =e^{-\left(\frac{{\left(x-n_{1}a\right)}^{2}}{\sigma_{x_{r}}{\;}^{2}}+\frac{{\left(y-n_{2}a\right)}^{2}}{\sigma_{y_{r}}{\;}^{2}}\right)}e^{-j2\pi \frac{\left(m_{1}bx/L_{1}+m_{2}by\right)}{L_{2}}}, \end{array}$$

where *L*_1_ and *L*_2_ are the total number of voxels in the slice in *X* and *Y* directions, *x* and *y* are space coordinates within the slice, *a* and *b* are the magnitudes of the isotropic shifts in the spatial and frequency domains, respectively, *n*_1, 2_ and *m*_1, 2_ are the indices of the shifts in the position and frequency domains, respectively, σ_*x*_*r*__ and σ_*y*_*r*__ are variance parameters of the *r*-th window, and *r*∈0…*R*−1 with R denoting the number of windows. Let **G** be the Gabor matrix, whose columns are generated using all possible shift values for *a* and *b* for all *R* windows with every *x* and *y* represented in each column. The filter bank coefficients **c** are obtained by convolving each volume slice-by-slice with **G**. The same **G** matrix is used for all volumes. An example of a representative Gabor filter output for a stroke lesion is shown in Figure 3.

#### 2.3.3. Gabor Window Parameter Optimization

For the initial Bayesian classification, only two classes, non-lesion and lesion, are used. In order to optimize the Gabor window parameters, the remapping window remains fixed while the analysis window *g*_*r*_[·] is varied over the parameters (σ_*x*_*r*__ and σ_*y*_*r*__) aiming to maximize the distance between the non-lesion and lesion classes. More formally, let {**f**_*t*_} and {**f**_*h*_} be the sets of voxels belonging to the stroke lesion class and the non-lesion class, respectively, as suggested in Subbanna et al. (16). The corresponding lesion coefficients **c**_*t*_ in the time-frequency domain are obtained by a convolution of the set of Gabor filters, with the center of the Gabor mask aligning with the stroke voxel, whose texture is being computed. Similarly, the non-lesioned tissue coefficients **c**_*h*_ are obtained by convolution with the Gabor filters, with the center of the Gabor mask aligning with the healthy voxels, whose textures are computed, at the non-lesion voxels. For maximum separation between the lesion and the non-lesion classes, the following formula is optimized:

(3) $$\arg\underset{\sigma_{x},\sigma_{y}}{\max}\sum_{j,k}\text{​}\text{​}|c_{j}-c_{k},|\;\forall c_{j}\in \{c_{t}\},\;\forall c_{k}\in \{c_{h}\}$$

where σ_*x*_ and σ_*y*_ are the vectors containing the *R* values of σ_*x*_*r*__ and σ_*y*_*r*__ for the different spreads in the *x* and the *y* directions. Practically, this optimization problem is solved using a graph cut approach (27).

#### 2.3.4. Customized Markov Random Fields

The principal purpose of this stage is to refine the initial voxel-based classification by considering the local observations and contexts. The MRF used for this purpose is specifically designed to not only use intensities at the voxel level as suggested in the standard Potts model (28), which simply uses the observations at the voxel-level and just pairwise voxel cliques, but also to model the intensity differences in the neighborhood and the textures of the voxels, to preserve lesion boundaries correctly.

The label *C*_*i*_ of voxel *i* can be inferred probabilistically from the intensity vector **I**_*i*_, texture vector **T**_*i*_, and the intensity difference vector **ΔI**_*N*_*i*__, which describes the intensity difference between the voxel and its neighbors in the cliques. The cliques, in this case, are the sets of voxels in the defined neighborhood that share either an edge or a vertex. Thus, for an 8 voxel neighborhood, there are 2 voxel, 3 voxel, and 4 voxel cliques. The probability of the label at the voxel *i* can be inferred from:

(4) $$\begin{array}{l} P(C_{i}|I_{i},T_{i},\Delta I_{N_{i}})=\sum_{C_{N_{i}}}P(C_{i},C_{N_{i}}|I_{i},T_{i},\Delta I_{N_{i}})\\ \;=\sum_{C_{N_{i}}}\frac{P(\Delta I_{N_{i}},I_{i},T_{i}|C_{N_{i}},C_{i})P(C_{N_{i}},C_{i})}{P(\Delta I_{N_{i}},I_{i},T_{i})}\\ \;\propto \sum_{C_{N_{i}}}P(\Delta I_{N_{i}}|I_{i},T_{i},C_{N_{i}},C_{i})P(T_{i}|I_{i},C_{N_{i}},C_{i})\\ \;P(I_{i}|C_{N_{i}},C_{i})P(C_{N_{i}}|C_{i})P(C_{i})\\ \;\approx \sum_{C_{N_{i}}}P(\Delta I_{N_{i}}|C_{N_{i}},C_{i})P(T_{i}|C_{i})P(I_{i}|C_{i})\\ \;P(C_{N_{i}}|C_{i})P(C_{i}) \end{array}$$

Equation 4 is obtained from the preceeding equation using Bayes rule and assuming that *P*(**ΔI**_*N*_*i*__∣**C**_*N*_*i*__, **T**_*i*_, *C*_*i*_, **I**_*I*_)=*P*(**ΔI**_*N*_*i*__∣**C**_*N*_*i*__, *C*_*i*_), *P*(**T**_*i*_∣**I**_*i*_, **C**_*N*_*i*__, *C*_*i*_)=*P*(**T**_*i*_∣*C*_*i*_), and *P*(**I**_*i*_∣**C**_*N*_*i*__, *C*_*i*_)=*P*(**I**_*i*_∣*C*_*i*_). In Equation 4, *P*(*C*_*i*_) is the prior probability of class *C*_*i*_ as determined by the registered atlas tissue map, *P*(**I**_*i*_∣*C*_*i*_) is the likelihood of *C*_*i*_ given **I**_*i*_, *P*(**T**_*i*_∣*C*_*i*_) models the likelihood of *C*_*i*_ given the texture **T**_*i*_, *P*(**ΔI**_*N*_*i*__∣**C**_*N*_*i*__, *C*_*i*_) models the intensity difference between voxel *i* and its neighbors in the clique, given the neighboring classes, and *P*(**C**_*N*_*i*__∣*C*_*i*_) models the probability of transition between *C*_*i*_ and **C**_*N*_*i*__. **T**_*i*_ at every voxel are computed using the set of Gabor windows described in 2.3.2, centered at voxel *i*. To infer the class labels, the energies at all voxels *i, i*∈0, …, *Q*−1 have to be minimized simultaneously. Assuming that Gibbs sampling assumption holds true (28), the MRF energy equation for the energy at voxel *i* is given by:

(5) $$\begin{array}{l} U(C_{i}|I)=-\log P(I_{i}|C_{i})-\log(T_{i}|C_{i})-\log P(C_{i})\\ \;-\sum_{k\in \text{Cliques}(N_{i})}\text{​}\text{​ }\sum_{j\in k}\left(\log P(\Delta I_{i,j}|C_{i},C_{j})-\alpha m(C_{j},C_{i})\right), \end{array}$$

where **I** is the intensity vector for all voxels *i*∈0, …, *Q*−1, *k* indexes over all the possible clique sizes in *N*_*i*_, and *j* indexes over all possible clique combinations of size *k*, *m*(**C**_*j*_, *C*_*i*_) is the potential associated with the spatial relationship between *C*_*i*_ and the vector of classes in the *j*th clique **C**_*j*_, and α is a weighting parameter used to handle the differences between inter-slice and intra-slice distances. Specifically, α is computed using the ratio of the intra-slice and inter-slice distances.

#### 2.3.5. Training and Classification

The neighborhood considered in this work includes 8 in-plane neighbors and the corresponding voxels in the slices above and below. All possible cliques, i.e., all 2, 3, and 4 voxel cliques, are considered. Gaussian distributions were used to model the non-lesion and lesion tissue classes. Similarly, intensity difference likelihoods are modeled using Gaussian distributions to describe the differences of lesions with other tissue classes. Finally, the texture coefficients are modeled by multivariate Gaussian distributions. The class transition probabilities are extracted from the frequency of co-occurrences of the classes in the training volumes. With the models for the tissue class intensities, tissue class intensity differences, and tissue class textures, and the statistics for the 2, 3, and 4 voxel cliques obtained from the training images, and the prior probabilities available from the registrations, the labels are inferred using Equation. 5. During classification, the combined energy of all voxels in Equation 5 is minimized using asynchronously updated iterated conditional modes (ICM) (29, 30) since the Bayesians usually give a good initial estimate for the subsequent optimization. The maximum iteration was set to 10, but convergence was typically achieved in less iterations.

### 2.4. Evaluation

Leave-one-patient-out evaluations were performed separately for each database. This did not include the optimization of the Gabor parameters, which were computed only once for each database using the datasets of another database and were kept constant to ensure consistency regarding the texture features but also to reduce the computational needs as this is a very time-consuming step. More precisely, the Gabor parameters for the classification of the SFB datasets were optimized using the datasets from the I-KNOW database and vice versa, while the SFB datasets were used for optimization of the Gabor window parameters required for the segmentation of the ISLES datasets. Given that the parameters for the initial classifications originate from different datasets acquired using different imaging parameters, this does not give advantage the proposed method.

The Dice score, which measures the overlap of the manual ground truth lesion segmentation and the automatic lesion segmentation was used as the primary outcome measure (for training and testing). Additional metrics calculated include the positive predictive value (PPV) and sensitivity of the classification, the ground truth and automatic lesion volume, as well as the average surface and Hausdorff distance. Additionally, Spearman's rank-order correlation was used to compare the automatically derived lesion volumes with the corresponding ground truth lesion volumes.

Furthermore, the results of the proposed method were also compared to the lesion segmentation results of two state-of-the-art lesion segmentation methods that performed best in the ISLES 2015 challenge. The first technique (31) is a method based on convolutional neural networks, while the second approach (32) is a technique based on fuzzy C-means clustering to obtain an initial classification, which is refined using a three-phase level set segmentation technique. Of note, neither of the implementations can be assumed to be exact replica of the original implementation. The comparison techniques were re-implemented based on the code available from the authors and other described filter steps required but not part of the available source code. In case of the lesion segmentation using convolutional neural networks, the general network setup was not modified but only re-trained and tested using the same leave-one-patient-out evaluation scheme as used for evaluation of the proposed method. In case of the fuzzy c-means and level-set method, only the fuzzy c-means method was optimized for this evaluation while the parameters for the level-set method described by the authors were not modified.

## 3. Results

Visual analysis of the bias field correction results resulted in exclusion of six datasets from the I-KNOW database and two datasets from the SFB database. Computationally, the proposed method requires between 20 and 30 min for lesion segmentation in the I-KNOW and SFB datasets, depending on the number of iterations required for the algorithm to converge. Due to the high spatial resolution of the interpolated ISLES datasets, the computation time increases to 70 to 90 min. Likewise, the actual training of the classifier for lesion segmentation also depends on the training set and required about 50 to 60 min for the I-KNOW and the SFB data sets, and about three hours for the ISLES data set. However, the training of the Gabor filter parameters is the computationally most expensive step and requires multiple hours. However, both training procedures only need to be computed once for lesion segmentation of new datasets.

### 3.1. Qualitative Results

**Figure 4:**
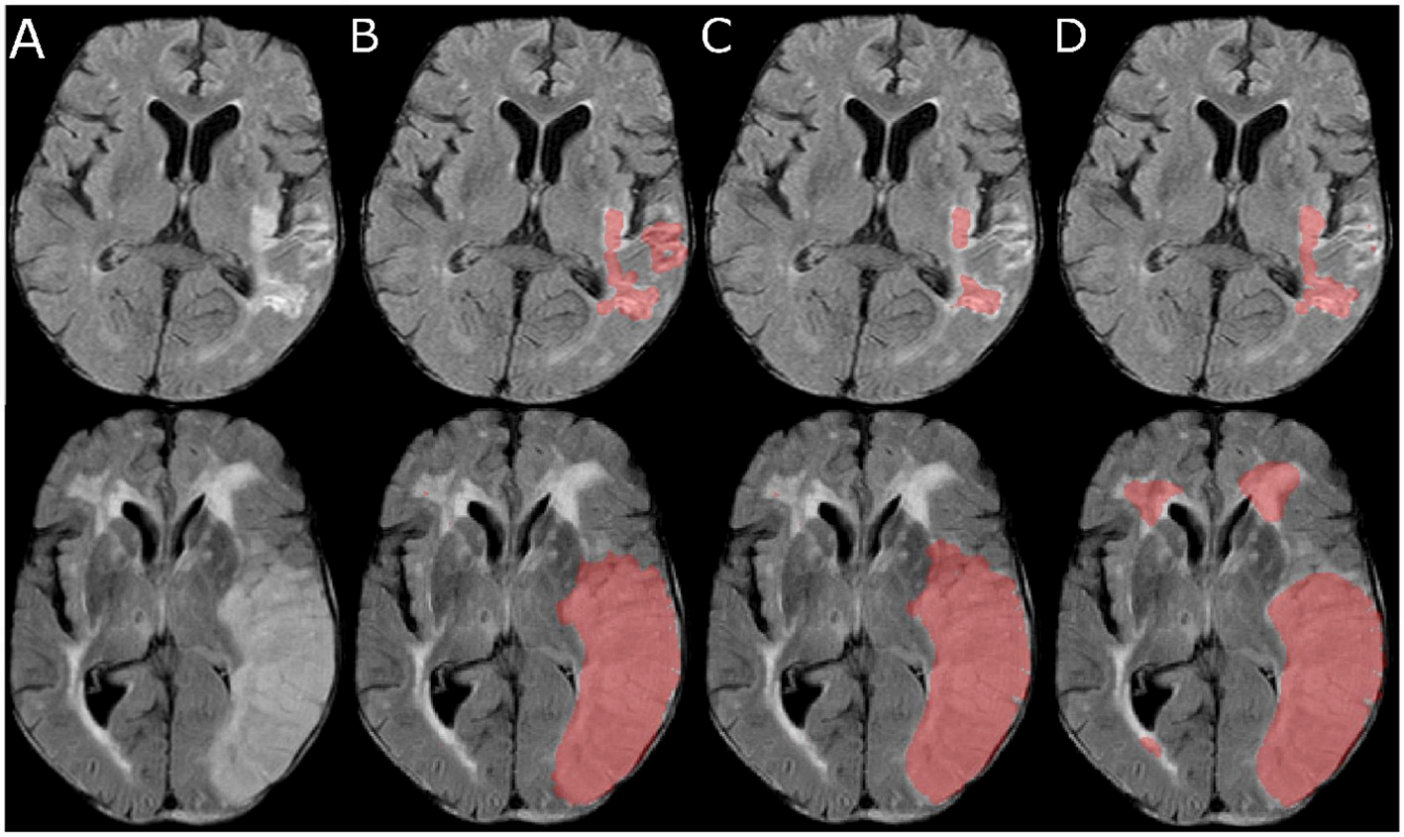
Selected slices **(A)** from two FLAIR MRI datasets (top and bottom) and corresponding automatic lesion segmentations generated using the proposed method **(B)**, the convolutional neural network approach **(C)**, and the fuzzy C-means clustering and level-set method **(D)**.

The qualitative results for two selected patients are shown in Figure 4. Lesions of two different sizes and shapes, in different regions were selected for this. Overall, it can be seen that the proposed approach successfully identifies and segments all lesions independent of the size and location, which includes even small lesions with complex, non-compact shapes (see Figure 4 top), while at the same time not segmenting periventricular lesions, which was one of the problems observed for the comparison method employing a combination of fuzzy C-means clustering and a three-phase level set segmentation technique (see Figure 4 bottom).

### 3.2. Quantitative Results

Overall, the quantitative results (Table 1) support the findings based on the qualitative results described above. Generally, the segmentation evaluation metrics for the proposed lesion segmentation method are well in line with the results from the ISLES challenge generated using a separate testing set, in which the winning algorithm achieved a mean Dice coefficient of 0.59, mean average surface distance of 5.95 mm, and average Hausdorff distance of 37.88 mm (19). However, the highest ranked method achieving these results employed regional random forests based on the available multi-modal MRI datasets, while the method proposed in this work is using only single-channel FLAIR MRI datasets.

**Table 1:** Quantitative results (mean ± standard deviation) of the proposed lesion segmentation method for the three databases are shown in the table.

**Dataset**	**Images**	**D**	**PPV**	**Sensitivity**	**ASD**	**HD**
SFB	23	0.621 ± 0.26	0.551	0.615	6.8 ± 8.26	32.15 ± 15.35
I-KNOW	102	0.583 ± 0.236	0.742	0.464	7.39 ± 9.32	36.23 ± 27.32
ISLES	26	0.544 ± 0.28	0.422	0.594	8.91 ± 10.15	35.34 ± 31.94
Wtd. Avg.	151	0.582 ± 0.25	0.658	0.509	7.56 ± 9.29	35.38 ± 25.97

*D, Dice; PPV, Positive predictive value; the sensitivity; ASD, Average surface distance (in mm); HD, Hausdorff distance (in mm) are presented*.

Furthermore, the average quantitative results revealed a volumetric lesion undersegmentation for the proposed method in the I-KNOW database (see Table 2), while the other two databases lead to rather comparable volumes for the automatically and manually segmented lesions. Taking all segmentation evaluation metrics into account, the best segmentation results were achieved for the SFB database, followed by the I-KNOW database, while the segmentation of the ISLES datasets led to the overall worst results. In line with these results, Spearman's rank-order correlation comparing the automatically extracted lesion volumes with the manually segmented lesion volumes was lowest but still very strong for the ISLES datasets (*r* = 0.74) and similar for the I-KNOW (*r* = 0.84) and SFB (*r* = 0.81) datasets. However, the overall correlation of *r* = 0.81 across all databases is very strong.

**Table 2:** *V*_*man*_ = Volume of the manual lesion segmentation (in mL), *V*_*aut*_ = Volume of the automatic lesion segmentation (in mL), and the Spearman correlation coefficient for volumetric correlation for all datasets for our proposed algorithm.

**Dataset**	**Images**	***V_man_***	***V_aut_***	**Spearman coefficient**
SFB	23	23.82 ± 26.18	23.36 ± 23.23	0.81
I-KNOW	102	42.95 ± 57.6	26.82 ± 41.37	0.84
ISLES	26	43.28 ± 57.64	52.08 ± 41.42	0.74
Wtd. Avg.	151	40.09 ± 51.83	30.65 ± 40.07	0.81

**Figure 5:**
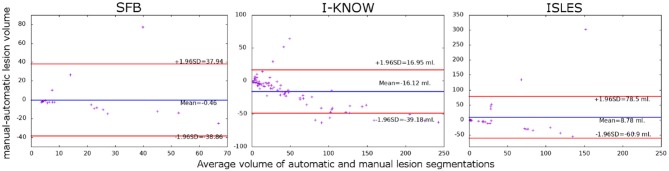
Bland-Altman plots showing the relationship between the difference and mean of the manually and automatically segmented stroke lesions for the three databases used for evaluation.

The corresponding Bland-Altman plots (see Figure 5) generally support the previously described findings but also show that the proposed method increasingly underestimates the volume of larger stroke lesions compared to the manual ground truth segmentation. This tendency toward underestimated lesion volumes is masked in the average lesion volumes in the SFB and ISLES database due to 1 and 2, respectively, considerably over-segmented lesions, which were relatively less frequent in the I-KNOW database. In all cases, these few outliers originated from small lesions that were in close vicinity to other larger white matter lesions, which are difficult to separate even for human experts.

Small lesions are often falsely segmented by automatic methods if only FLAIR datasets are used for this purpose. Connected component analysis using a 26-neighborhood analysis of the manually segmented lesions in all datasets from the three databases identified a total of 483 lesion components that are smaller than 1 ml. These small lesions were further analyzed to investigate the performance of the proposed algorithm to segment small lesions components in more detail. Therefore, a small lesion was classified as a true-positive segmentation if at least three voxels within the lesion are segmented by the proposed automatic method. Likewise, a lesion component segmented by the automatic method was defined as a false-positive if the corresponding manual lesion segmentation contains less than three segmented voxels in that area. Based on these definitions, the proposed method segmented 258 of the 483 lesions correctly while falsely segmenting 81 lesions smaller than 1 ml.

### 3.3. Comparison to State of the Art Techniques

The quantitative results of the comparison of the proposed method to the two comparison methods are shown in Table 3. Here, it can be seen that the proposed technique leads to improved lesion segmentation results compared to the other two automatic lesion segmentation methods with an improvement of around 8% compared to the method using a convolutional neural network, which in turn performed better than the combined fuzzy C-means and level-set lesion segmentation method, which is also in line with the results from the ISLES challenge 2015.

**Table 3:** The Dice scores and standard deviation for the three automatic lesion segmentation methods (CNN, convolutional neural network; FCM, Fuzzy C-Means).

**Database**	**Images**	**Proposed method**	**CNN (31)**	**FCM and level sets (32)**
SFB	23	0.621 ± 0.260	0.572 ± 0.230	0.534 ± 0.280
I-Know	102	0.583 ± 0.236	0.535 ± 0.301	0.521 ± 0.288
ISLES	26	0.544 ± 0.280	0.541 ± 0.270	0.527 ± 0.299
Wtd. Avg.	151	0.582 ± 0.250	0.541 ± 0.272	0.524 ± 0.287

A paired t-test comparing the Dice scores using the entire set of 151 datasets revealed that the proposed method performs significantly better compared to the convolutional neural network lesion segmentation method (*p* = 0.046) and the combined fuzzy C-means and level-set lesion segmentation method (*p* = 0.041).

Using the same definitions for small lesions, revealed that the convolutional neural network lesion segmentation method was able to identify a slightly smaller number of true-positive lesions (*n* = 239) but considerably more false-positive lesions (n = 131) compared to the proposed method (*n* = 258/*n* = 81). The combined fuzzy C-means and level-set lesion segmentation method identified considerably less true-positive small lesions (*n* = 209) but performed equally as well considering false-positive small lesions (*n* = 84) compared to the proposed method.

In a final evaluation, the generalizability of the three segmentation methods was investigated. Therefore, all three methods were trained/optimized using all datasets from the I-KNOW database, which was selected for this purpose due to the number of datasets and rather large data variability resulting from the multi-center image acquisition. The trained models were then applied to the SFB datasets. The ISLES were excluded from this analysis because these datasets were only available registered and resampled to the corresponding T1-weighted MRI datasets, which introduces interpolation artifacts. For all three methods, the drop in Dice score comparing the manual with the automatically segmented lesions was comparable for the proposed method and the method using the convolutional neural network and considerably higher for the fuzzy C-means and level-set method. More precisely, the proposed method achieved a Dice score of 0.56±0.26, the convolutional neural network a Dice score of 0.51±0.25, and the fuzzy C-means and level-set method a Dice score of 0.44±0.3.

## 4. Discussion and Conclusion

This work presented a novel method for the segmentation of sub-acute stroke lesions from uni-modal FLAIR MRI datasets using modified Markov random fields employing intensity, intensity variation, and texture features.

Overall, the quantitative results are in the upper range of previously presented methods. However, it needs to be highlighted that the results of most of these methods are not directly comparable as different databases were used for the development and evaluation of the methods. The highest ranking method of the ISLES challenge of 2015 achieved an average Dice similarity coefficient of 0.59, mean average surface distance of 5.95 mm, and average Hausdorff distance of 37.88 mm (19), which is in the range of the average Dice similarity coefficient of 0.582, mean average surface distance of 7.56 mm, and average Hausdorff distance of 35.38 mm achieved by the proposed method in this work. However, it has to be highlighted that the ISLES datasets consisted of multi-modal MRI sequences including T1-weighted, T2-weighted, diffusion-weighted, and FLAIR MRI datasets. The inclusion of such multi-modal imaging information can help to improve the segmentation accuracy, but in this work, only FLAIR datasets were considered for the lesion segmentation. As noted above, the pre-processing of such a multi-modal dataset can be very challenging and the whole range of these sequences is not always available in typical stroke follow-up studies. For this reason, the method presented in this work was developed and evaluated only based on single-channel FLAIR MRI datasets, which are typically part of any stroke follow-up MRI protocol. In this respect, the quantitative results achieved by the proposed method can be considered very good. In addition, it should be noted that the lesion segmentation approach presented in this paper is highly flexible and an integration of multi-modal MRI datasets for lesion segmentation is possible.

The average Dice similarity metric was lowest for the ISLES database compared to the other two databases used for evaluation, whereas the average Dice similarity value was better for the SFB dataset compared to the I-KNOW database. One potential reason why the Dice score was best for the SFB database is that all of these datasets were acquired on the same scanner with the same imaging parameters so that all of these images are much more similar compared to the two other two databases, which consist of datasets acquired in multiple centers. Furthermore, it should be highlighted that the FLAIR MRI datasets in the SFB database were acquired using a 3T MRI scanner, which results in a better signal-to-noise ratio compared to datasets acquired on 1.5T scanners as used for the other two databases. As a result of this, the volumes of the automatically segmented lesions are very similar to the volumes of the manual ground truth segmentation with a very strong correlation.

A potential reason for the worse quantitative results (Dice and lowest overall correlation compared to manual segmentations) obtained for ISLES datasets compared to the other two database could be that the FLAIR MRI datasets were not available in the original imaging space but only registered and resampled to the corresponding T1-weighted image, which comes at the expense of interpolation-related artifacts, which, for example, affects the Gabor texture feature calculation making it not comparable to the other two databases. For this reason, a pooling of the three databases for a combined leave-one-out evaluation was not conducted. This problem likely also caused the volumetric overestimation of the lesions segmented using the proposed method compared to the manual segmentations found for this database.

**Figure 6:**
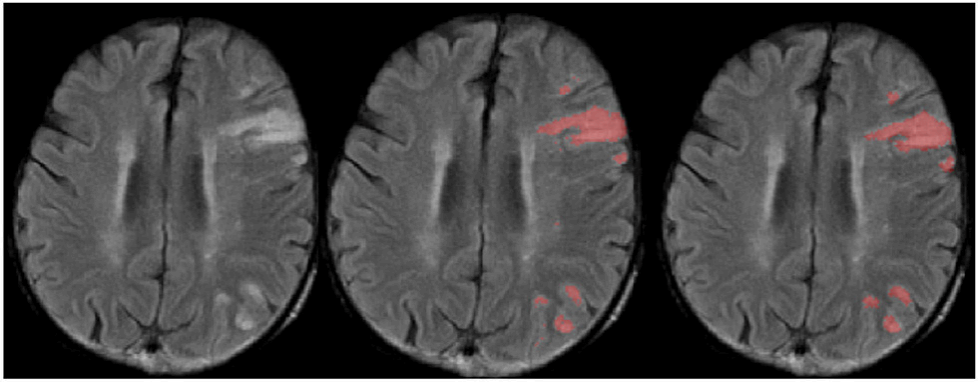
Selected slice from a FLAIR MRI dataset with multiple lesions **(Left)** and corresponding manual **(Center)** and automatic lesion segmentation **(Right)**.

Another benefit of the proposed method is that it can detect multiple stroke lesions with higher accuracy, while at the same time small white matter hyperintensities not part of the ischemic stroke lesion are correctly segmented less often by the proposed method compared to the other two approaches tested (see Figure 6).

However, this benefit also comes along with the drawback that the automatic lesion segmentations seem to be underestimated at the border leading to the reduced volumes compared to the true manually segmented lesion volumes. As the lesion volume increases, the volumetric underestimation is also increasing, which is related to the increased surface-area-to-volume ratio of complex structures. Due to this volumetric underestimation, the proposed method is not yet optimally suited to be used to extract the lesion volume as a clinical endpoint using FLAIR datasets only. However, the comparably good results achieved in this study suggest that it represents a good basis for further refinement to achieve this goal. A potential technical improvement to the method to overcome this issue is non-uniform weighting of contrasts and textures based on the location of the voxel with respect to the lesion boundaries. Texture features are more characteristic for the lesion core, while the contrasts are more useful at the boundaries.

Another limitation is that the proposed method was not validated using a completely independent validation set. Training of all three methods with the I-KNOW database and application to the SFB datasets resulted in a similar drop in Dice score for all three methods, which might raise concerns regarding the generalizability. However, it needs to be highlighted that the SFB datasets were acquired on a 3T MRI scanner while the datasets of the I-KNOW database were acquired on various 1.5T MRI scanners, which likely explains the drop in Dice score. Thus, another multi-center dataset would be required for a true validation and investigation of the generalizability abilities. Within this context, it should also be mentioned that not all hyper-parameters of the two comparison methods, e.g., the general deep neural network architecture or the exact level-set parameters, were optimized again in this work but taken directly from the available source code corresponding to the methods achieving the best results in the ISLES challenge. Thus, the results of the two comparison methods could be further improved by further optimization of all potentially relevant parameters.

In conclusion, the proposed lesion segmentation method leads to robust segmentation results of lesions in follow-up FLAIR dataset and could prove valuable for lesion volume estimations in future stroke trials.

## Author Contributions

NS: study design, image processing, data analysis, drafting the manuscript, and revising it critically. DR and TA: image processing and critical revision of the manuscript. BC, GT and JF: data acquisition and critical revision of the manuscript. NF: study design, image processing, data analysis, drafting the manuscript and revising it critically.

### Conflict of Interest Statement

The authors declare that the research was conducted in the absence of any commercial or financial relationships that could be construed as a potential conflict of interest.
